# Rapeseed Biofumigation Combined with Biochar Mitigates Continuous-Cropping Obstacles in *Chrysanthemum* via Soil Improvement, Rhizosphere Microbial-Community Modification, and Wilt Disease Reduction

**DOI:** 10.3390/microorganisms14102383

**Published:** 2026-10-18

**Authors:** Zhong Yao, Gaochuan Shi, Kaipeng Xi, Yurong Qiu, Mingang Tao, Fengfeng Shen, Wei Wang, Zhen Liu

**Affiliations:** 1 Institute of Cotton Research, Shanxi Agricultural University, Yuncheng 044000, China; 2 Yuncheng Agriculture and Rural Bureau, Yuncheng 044000, China

**Keywords:** rapeseed biofumigation, biochar, chrysanthemum, soil improvement, microbial community, wilt disease

## Abstract

Long-term monoculture degrades soil ecological conditions and is associated with severe wilt disease in *Chrysanthemum morifolium*. The effects of combining rapeseed biofumigation and biochar to alleviate these continuous cropping obstacles remain poorly understood. A field experiment compared a control (CK), rapeseed biofumigation alone (R), and biofumigation combined with corn-straw biochar (RB) in soil under eight years of continuous chrysanthemum cultivation. Soil properties, enzyme activities, wilt disease severity, chrysanthemum yield, and rhizosphere microbial communities were assessed. Compared with CK, RB significantly increased available phosphorus (+40.7%), available potassium (+16.4%), total nitrogen (+4.9%), cation exchange capacity (+8.6%), selected enzyme activities, plant height, and flower yield (*p* < 0.05). The disease index decreased from 8.67 in CK to 2.67 under R and 2.33 under RB, with both reductions significant but no significant difference between R and RB. Both treatments altered microbial community composition, reduced bacterial richness, and enriched MND1; RB additionally reduced bacterial diversity, enriched Acidobacteriota, and decreased the abundances of Firmicutes and Bacteroidota relative to CK. Exploratory Mantel tests indicated that available phosphorus and cation exchange capacity were associated with variation in rhizosphere microbial community structure. Overall, RB improved selected soil fertility parameters and flower yield, accompanied by microbial community changes and lower wilt severity. Broader application requires multi-site and multi-season validation.

## 1. Introduction

Obstacles to continuous cropping create a critical bottleneck in the large-scale and sustainable cultivation of chrysanthemum flowers [1,2,3]. As a characteristic economically valuable medicinal and edible flower in China, *Chrysanthemum morifolium* Ramat. is widely processed into herbal tea, with substantial market demand and economic value [1]. Limited arable land resources and specialized contiguous monoculture patterns have contributed to the widespread use of long-term continuous chrysanthemum monocropping [1]. Long-term monoculture can lead to soil physicochemical degradation, nutrient imbalance, and rhizosphere microecological disruption, accompanied by the accumulation of soil-borne pathogens [4,5,6,7]. These adverse changes can impair plant growth, reduce flower yield and quality, and are associated with frequent outbreaks of wilt disease, severely hindering the development of the tea chrysanthemum industry [1,2,3]. Previous studies have reported that wilt disease is a devastating soil-borne disease in chrysanthemum and can be particularly severe in continuously cropped fields [1,2]. Long-term chemical fungicide application carries multiple risks, including accumulation of pesticide residues, disruption of soil fungal communities, and the development of fungicide resistance [8]. Therefore, there is an urgent need to develop green, efficient, and sustainable soil amelioration and disease management technologies to reduce wilt disease severity in tea chrysanthemum and to alleviate obstacles to continuous cropping.

Biofumigation, an environmentally friendly biological soil-remediation strategy, has attracted extensive attention in recent decades. Its core mechanism relies on the abundant glucosinolates contained in cruciferous plant tissues, which degrade to release volatile isothiocyanates (ITCs) that can suppress soil-borne pathogens [9,10]. *Brassica juncea* is regarded as one of the most promising biofumigation materials, owing to its high glucosinolate concentration, high biomass production, and strong environmental adaptability [11]. Rapeseed biofumigation has been reported to improve the physicochemical properties of continuously cropped soil, elevate available soil nutrient pools, and reduce the population density of pathogenic fungi, with associated reductions in chrysanthemum wilt disease incidence [12]. Within continuous chrysanthemum monoculture systems, rapeseed biofumigation alone or combined with organic amendments such as wheat bran achieved 33.65–55.70% control efficacy against chrysanthemum wilt disease, while markedly promoting chrysanthemum growth [12]. Nevertheless, some soil microbial responses to biofumigation may diminish over time [13,14], and the durability of its effects requires further evaluation.

Biochar, characterized by its porous architecture, large specific surface area, and strong adsorption capacity, has been used as a soil amendment in recent years. Biochar can improve soil fertility by enhancing nutrient retention, elevating the cation exchange capacity (CEC), and providing favorable microhabitats for soil microorganisms [15]. Biochar can reshape soil microbial assemblages [16,17] and favor potentially beneficial microbial taxa [18]. Owing to its strong adsorption capacity, biochar may alter the adsorption and dissipation of fumigant compounds, potentially modifying their availability and biological effects [19]. Although both practices show promise for restoring soil health for chrysanthemum production, their integrated effects on wilt disease reduction in chrysanthemum, microbial community assembly, and soil ecological processes remain poorly understood, particularly in tea chrysanthemum planting systems.

Wenxi County (Yuncheng, Shanxi) is a major contiguous tea chrysanthemum-growing area in northern China. Long-term monocropping here has been associated with severe wilt disease and declining soil fertility, creating an urgent need for locally suitable green soil amendments. Over more than a decade, our research group has examined biofumigation technologies for alleviating obstacles to continuous cropping. We have bred the high-glucosinolate, high-biomass *Brassica juncea* ‘Jinhuangjie’ and have established a mature disease-control system effective against wilt disease in watermelon and damping-off and Verticillium wilt in cotton [20,21]. Our core research was formally recognized as a national scientific achievement in 2017 in China, and the regional biofumigation protocol was listed as a major extension technology across Shanxi in 2023. Building on this established framework, the present field experiment was conducted on eight-year continuously monocropped tea chrysanthemum brown soil, using ‘Jinhuangjie’ rapeseed biofumigation combined with corn-straw biochar. The three treatments were a blank monocropping control (CK), rapeseed biofumigation alone (R), and biofumigation co-application with corn-straw biochar (RB). This study aimed to evaluate whether biochar supplementation could further enhance the effects of rapeseed biofumigation on soil properties, rhizosphere microbial communities, chrysanthemum yield, and wilt disease severity in a continuous monocropping system. We further examined the associations among soil nutrients, enzyme activities, and rhizosphere microbial community composition. The findings will provide field-based theoretical support and practical amelioration strategies for the green mitigation of barriers to continuous cropping in chrysanthemum and to improve the ecological management of soil-borne diseases.

## 2. Materials and Methods

### 2.1. Experimental Materials

The field experiment was conducted in Wenxi County, Yuncheng City, Shanxi Province, China (35°29′ N, 111°12′ E, elevation 495 m a.s.l.). The study area has a warm temperate continental monsoon climate, an annual average temperature of 12.7 °C, and annual precipitation of 506.55 mm. The test soil was classified as brown soil, and the experimental field had undergone eight consecutive years of continuous chrysanthemum monocropping before the trial.

The green manure used for biofumigation was from *Brassica juncea* ‘Jinhuangjie’, a high-glucosinolate cultivar specifically bred for biofumigation applications. Its shoot and root glucosinolate concentrations range from 60.82 to 70.64 μmol·g^−1^. Corn-straw biochar was purchased from Henan Lize Environmental Protection Technology Co., Ltd. (Zhengzhou, China). The biochar exhibited a pH of 9.46 and contained 42.21% organic carbon, 8.34% total nitrogen, 2.31% total phosphorus, 16.12% total potassium, and 7.23% ash. The chrysanthemum cultivar used was *C. morifolium* Ramat. ‘Ruhe’.

### 2.2. Experimental Design

The field experiment adopted a randomized complete block design with three treatments (CK, R and RB) and three biological replicates per treatment, with each plot covering 30 m^2^. The CK group represented the continuous-cropping control without rapeseed biofumigation or biochar amendment, the R group received rapeseed biofumigation alone, and the RB group received rapeseed biofumigation with corn-straw biochar application.

For the R treatment, *B. juncea* ‘Jinhuangjie’ was sown in mid-October 2023. Based on the locally promoted biofumigation technical standard of Shanxi Province and on previous laboratory research, the seeding rate was set at 1 kg per 666.7 m^2^ (equivalent to 1.5 × 10^−3^ kg·m^−2^). In April 2024, when the aboveground fresh biomass of rapeseed reached 5–6 kg·m^−2^, the entire rapeseed plants were mechanically chopped and incorporated into the top 15 cm soil layer via rotary tillage. The field was irrigated with water at a volume of 450 m^3^·ha^−1^, then immediately covered with polyethylene plastic film for 15 d of sealed fumigation. After film removal, the soil was ventilated for 7 d before the chrysanthemum cuttings were transplanted.

For the RB treatment, corn-straw biochar was uniformly incorporated into the soil at a dosage of 3 kg·m^−2^ (i.e., 30 t·ha^−1^) simultaneously with rapeseed residue incorporation during biofumigation implementation. The application rate was determined based on our earlier pot experiments and field research on biochar amendment in continuous-cropping soil [22]. All other field management practices were identical to those used for the R treatment. The CK group received identical routine field management without biofumigation or biochar addition. All plots were managed according to the local commercial chrysanthemum cultivation protocols throughout the growth cycle.

The sampling and investigation timelines were arranged according to the chrysanthemum growth stages (vegetative stage: cutting transplantation from April 2024 to late August 2024; reproductive stage: flower bud differentiation from late August 2024 to the end of flowering in September 2024). Rhizosphere soil collection and measurements of vegetative growth parameters were conducted on 27 June 2024, during the vegetative growth stage. Rhizosphere soil was collected from chrysanthemum plants in each plot via an X-shaped five-point sampling method. Briefly, individual chrysanthemum plants were carefully excavated using sterile small shovels. Loosely adhering bulk soil was removed by gently shaking the root system and manual brushing. Soil particles tightly attached to the root surface were defined as rhizosphere soil, which was carefully collected from root surfaces using sterile soft-bristle brushes and transferred into sterile 50 mL centrifuge tubes. Rhizosphere soils from five individual plants per plot were pooled and thoroughly mixed to form one composite sample per plot. All composite samples were immediately placed in an ice box for transport to the laboratory under low-temperature conditions for the analysis of soil physicochemical properties, enzyme activities, and microbial community composition. Field surveys of wilt disease severity were conducted on 23 July 2024 (vegetative growth stage), and the cumulative fresh flower yield was determined on 23 September 2024, after full flower harvest.

### 2.3. Determination of Soil Physicochemical Properties and Soil Enzyme Activities

All soil physicochemical indicators and enzyme assays were performed using the rhizosphere soil samples collected on 27 June 2024, during the vegetative growth period. Soil pH was measured using a glass electrode in a soil-water suspension. Alkali-hydrolyzable nitrogen (AN) was determined using the alkaline hydrolysis diffusion method. Available phosphorus (AP) was extracted using 0.5 mol·L^−1^ NaHCO_3_ and was quantified via molybdenum-antimony colorimetry. Available potassium (AK) was extracted using NH_4_OAc and was measured via flame photometry. Total nitrogen (TN), soil organic matter (OM), and CEC were determined using the Kjeldahl digestion method, potassium dichromate oxidation method, and ammonium acetate exchange method, respectively.

The activities of four key soil enzymes, catalase (S-CAT), sucrase (S-SC), urease (S-UE), and alkaline phosphatase (S-ALP), were measured using the corresponding commercial detection kits (Solarbio, Beijing, China), in accordance with the manufacturer’s standard operating procedures.

### 2.4. Assessment of Wilt Disease Severity

Field investigation of chrysanthemum wilt disease was carried out on 23 July 2024, during the vegetative growth stage. A five-point sampling strategy was adopted: five sampling points were selected in each plot, and five individual chrysanthemum plants were assessed at each sampling point to record disease severity grades. The disease index (DI) and disease control efficacy (CE) were calculated.

Disease severity was classified into five grades (0–4) based on plant symptom severity: 0, healthy plants with no visible wilt or yellowing symptoms; 1, slight chlorosis and leaf abscission on the basal leaves; 2, one-third to half of the leaves are yellow or wilted, accompanied by retarded plant growth; 3, half to three-quarters of the leaves exhibit yellowing and upward-spreading wilting symptoms; and 4, the plant is entirely wilted and dead.

DI was calculated as follows:

DI (%) = [Σ (number of plants in each grade × corresponding disease grade)/(total surveyed plants × highest disease grade)] × 100.(1)

CE (%) was calculated as follows:

CE (%) = [(DI_(CK)_ − DI_(treatment)_)/(DI_(CK)_)] × 100 (2)

where DI_(CK)_ and DI_(treatment)_ represent the disease indices of the control and treatment groups, respectively, and CK refers to the control.

### 2.5. Plant Growth Parameters and Yield Determination

Ten chrysanthemum plants were randomly selected from each plot on 27 June 2024 (vegetative growth stage) to measure the vegetative growth indicators. Plant height was measured using a soft measuring tape, and stem diameter was measured using a digital Vernier caliper (Yantai Greenery Tools Co., Ltd., Yantai, China). Relative leaf chlorophyll content (SPAD) was recorded using a portable Soil Plant Analysis Development-502 chlorophyll meter (Konica Minolta, Tokyo, Japan).

After full flower harvest on 23 September 2024, the fresh weight of chrysanthemum flowers from each plot was recorded to calculate cumulative yield, which was expressed in the unit of kg·m^−2^.

### 2.6. Soil DNA Extraction, PCR Amplification, and High-Throughput Sequencing

Total genomic DNA was extracted from 0.25 g fresh rhizosphere soil (sampled on 27 June 2024) using the TGuide S96 Magnetic Soil/Fecal DNA Kit (Tiangen Biotech Co., Ltd., Beijing, China), following the manufacturer’s instructions. DNA integrity was verified via electrophoresis on a 1.8% agarose gel, and the DNA concentration and purity were quantified using a NanoDrop 2000 spectrophotometer (Thermo Fisher Scientific, Waltham, MA, USA).

For bacterial community profiling, the V3–V4 hypervariable region of the 16S rRNA gene was amplified using the primer pair 338F (5′-ACTCCTACGGGAGGCAGCA-3′) and 806R (5′-GGACTACHVGGGTWTCTAAT-3′). For fungal community analysis, the internal transcribed spacer (ITS) region of the ribosomal RNA gene cluster was amplified using the primer set ITS1F (5′-CTTGGTCATTTAGGAAGTAA-3′) and ITS2R (5′-GCTGCGTTCTTCATCATGATGC-3′). The PCR amplification procedure was set as follows: initial denaturation at 95 °C for 5 min; 30 cycles of denaturation at 95 °C for 30 s, annealing at 50 °C for 30 s, and extension at 72 °C for 40 s; followed by a final extension step at 72 °C for 7 min. PCR products were purified using an Omega DNA Purification Kit (Omega Bio-Tek, Norcross, GA, USA), quantified on a Qsep-400 system (BiOptic Inc., New Taipei City, Taiwan, China), and sequenced on an Illumina NovaSeq 6000 platform (Biomarker Technologies Corporation, Beijing, China) using a 2 × 250 bp paired-end strategy. During the process, no-template controls were applied.

### 2.7. Bioinformatics and Statistical Analysis

The raw paired-end reads were quality-filtered, primer-trimmed, and denoised into amplicon sequence variants (ASVs) using the DADA2 algorithm [23] implemented in QIIME 2 (version 2020.6) [24]. Chimeric sequences were removed during the DADA2 denoising step. ASVs occurring fewer than 2 times across all samples were excluded as recommended by the platform (Feature Filter Threshold = 2) to remove potential sequencing artifacts. Taxonomic classification of representative ASV sequences was performed using the Naïve Bayesian classifier in QIIME 2 against the SILVA 138 database (bacteria) and the UNITE database (fungi), with a confidence threshold of 70%.

Alpha-diversity indices were calculated in QIIME 2. Soil physicochemical properties, enzyme activities, plant growth parameters, flower yield, and alpha-diversity indices were compared among treatments using one-way analysis of variance (ANOVA) followed by Duncan’s multiple-range test. Bray–Curtis dissimilarities were used for principal coordinates analysis (PCoA), permutational multivariate analysis of variance (PERMANOVA), and permutational analysis of multivariate dispersions (PERMDISP), with 999 permutations for both tests. When PERMDISP was significant, pairwise dispersion differences were assessed using Tukey’s honestly significant difference (HSD) test, and PERMANOVA results were interpreted alongside the dispersion results.

Differential abundance analysis at the phylum and genus levels was performed using ANCOM-BC2 on aggregated raw ASV counts. *p* values were adjusted using the Benjamini–Hochberg (BH) method and reported as *q* values. Robust differential abundance required an ANCOM-BC2 differential-abundance call, *q* < 0.05, and passed_ss = TRUE. The top 20 genera by mean relative abundance across all nine samples were defined as dominant genera. Pairwise log fold-change (LFC) estimates for R vs. CK, RB vs. CK, and RB vs. R were visualized as heatmaps; positive LFC values indicate higher estimated abundance in the first group.

Environmental variables included soil physicochemical properties (pH, OM, TN, AN, AP, AK, and CEC), enzyme activities (S-CAT, S-ALP, S-SC, and S-UE), and flower yield. CE was excluded from correlation analyses because it was derived from DI. Collinearity screening used pairwise Spearman correlations (|ρ| ≥ 0.80) and iterative variance inflation factor (VIF) screening combined with biological relevance, retaining TN, CEC, S-ALP, and yield for distance-based redundancy analysis (db-RDA) (all VIF < 10). AP was excluded from db-RDA because of collinearity with CEC but retained for exploratory univariate Mantel tests; its association was not interpreted as independent of other treatment-related variables.

Bray–Curtis-based db-RDA used 9999 permutations to test the overall model, constrained axes, and marginal variable effects; adjusted R^2^ was reported. Mantel tests used Spearman correlations between community Bray–Curtis dissimilarities and individual environmental Euclidean distances, with 9999 permutations. Spearman correlations were also calculated between dominant genera and environmental variables. Mantel *p* values were BH-adjusted across tested variables, and genus-environment Spearman *p* values were likewise BH-adjusted for multiple testing to control the false discovery rate (FDR); these adjusted values are reported as FDR-*p*. All correlation analyses were exploratory (*n* = 9) and potentially confounded by treatment-driven changes; they were interpreted as associations rather than causal relationships.

Bioinformatic analyses were completed on BMKCloud, and general statistical analyses and visualization were performed in R (version 4.4.2). DI was analyzed by ANOVA with treatment and block as factors, followed by Tukey’s HSD test (three plot-level replicates per treatment). CE was reported descriptively. The significance threshold was 0.05 for *p*, Tukey-adjusted *p*, *q*, or FDR-*p*, as applicable.

## 3. Results

### 3.1. Differences in Soil Physicochemical Traits and Soil Enzyme Activities Across Treatments

The effects of the different treatments on soil physicochemical properties are summarized in Table 1. No significant differences were detected among the treatment groups for soil pH, OM, and AN (*p* > 0.05). Relative to CK treatment, the R treatment markedly increased soil AP and CEC by 21.08% and 6.86%, respectively (*p* < 0.05), whereas AK and TN remained unchanged under R treatment (*p* > 0.05, Table 1). Compared with CK, RB significantly increased AP, AK, TN, and CEC by 40.71%, 16.35%, 4.90%, and 8.65%, respectively (*p* < 0.05, Table 1). Moreover, RB yielded substantially higher AP and AK concentrations than the R treatment (*p* < 0.05), and all four fertility-related indicators reached their maximum values under the RB treatment (Table 1).

**Figure 1:**
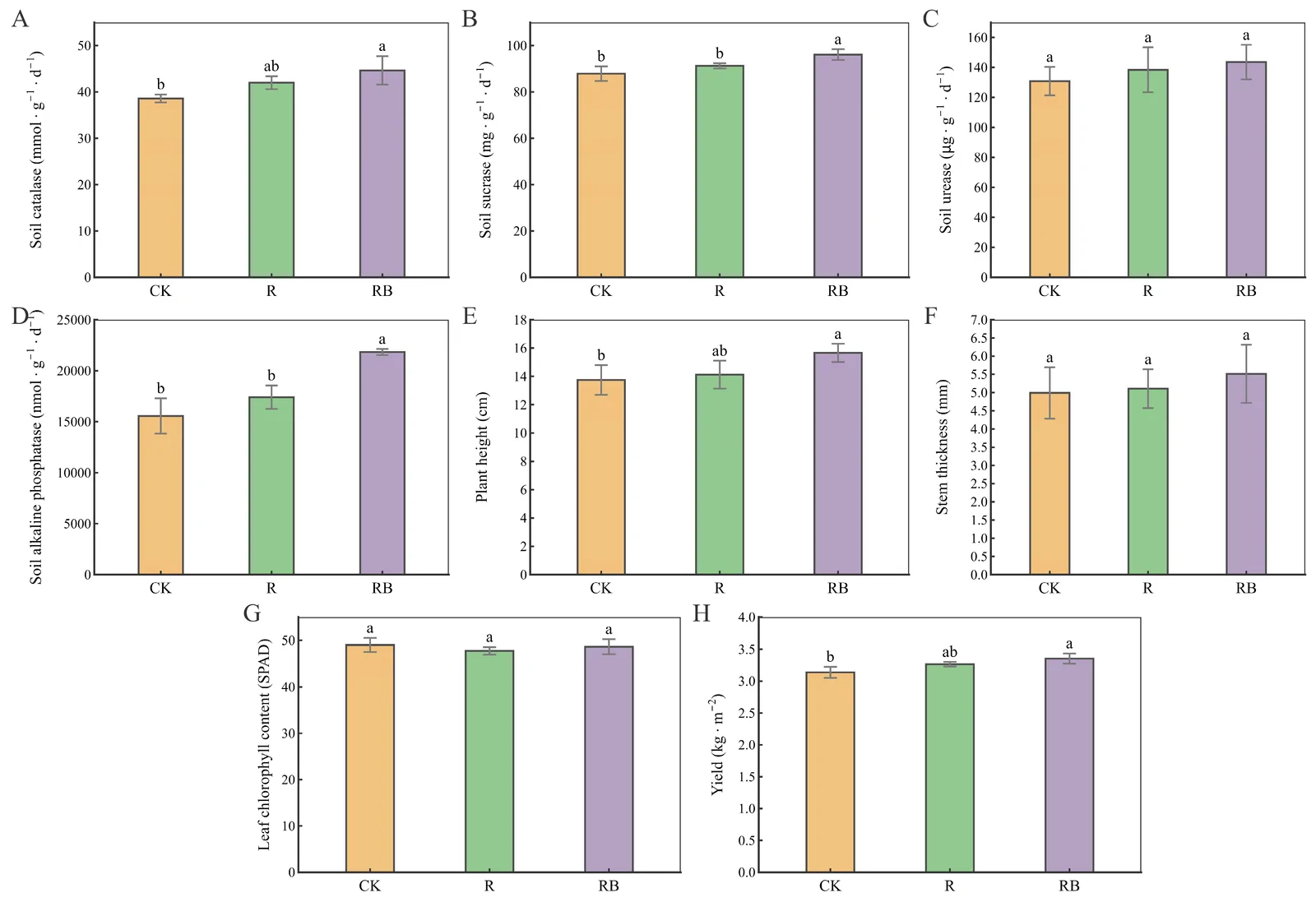
Effects of biofumigation combined with biochar amendment on soil enzyme activity and on chrysanthemum growth and yield. Levels of soil catalase (**A**), soil sucrase (**B**), soil urease (**C**), soil alkaline phosphatase (**D**), plant height (**E**), stem diameter (**F**), leaf chlorophyll content (**G**), and fresh flower yield (**H**) under three soil amendment regimes are shown. The data are shown as mean ± standard deviation (n = 3). Different lowercase letters indicate groups with significant inter-treatment differences (*p* < 0.05). CK, continuous-cropping control without rapeseed biofumigation or biochar amendment; R, rapeseed biofumigation alone; RB, rapeseed biofumigation with corn-straw biochar.

Distinct responses of soil enzyme activity to the different amendments were observed (Figure 1A–D). No significant differences in S-CAT (Figure 1A), S-SC (Figure 1B), or S-ALP (Figure 1D) activity were found between CK and R (*p* > 0.05). In contrast, RB significantly boosted S-CAT, S-SC, and S-ALP activities by 13.55%, 8.56%, and 28.72%, respectively, relative to the CK (*p* < 0.05). Further pairwise comparisons indicated that RB increased S-SC and S-ALP activities by 3.70% and 10.58%, respectively, relative to the R treatment (*p* < 0.05). S-UE activity did not differ among the three treatments (*p* > 0.05, Figure 1C).

### 3.2. Chrysanthemum Vegetative Growth and Flower Yield

Plant morphological traits and cumulative flower yields responded differently to the tested amendments (Figure 1E–H). Relative to the CK treatment, the RB treatment significantly enhanced plant height (Figure 1E) and total flower yield (Figure 1H) by 12.21% and 6.46%, respectively (*p* < 0.05). However, stem diameter (Figure 1F) and leaf SPAD values (Figure 1G) were not significantly different among the CK, R, and RB treatments (*p* > 0.05). The R treatment failed to induce significant improvements in any of the measured growth or yield parameters relative to the CK (*p* > 0.05, Figure 1E–H).

### 3.3. Wilt Disease Severity Under Different Soil Amendments

Both R and RB had significantly lower wilt disease indices than CK (Tukey-adjusted *p* = 0.00159 and 0.00129, respectively; Table 2). DI was 8.67 ± 1.15 under CK, 2.67 ± 0.58 under R, and 2.33 ± 0.58 under RB. No significant difference in DI was detected between R and RB (Tukey-adjusted *p* = 0.865). The corresponding descriptive CE values were 69.23 ± 6.66% for R and 73.08 ± 6.66% for RB.

### 3.4. Soil Microbial Alpha Diversity and Community Clustering Patterns

High-quality sequencing reads totaling 567,073 for bacteria and 507,880 for fungi were obtained after quality filtering; using the DADA2 denoising pipeline, these were resolved into 16,345 bacterial and 2958 fungal ASVs. Coverage values were at least 99.94% across all samples, indicating sufficient sequencing depth to adequately capture the amplicon diversity of the rhizosphere microbial communities (Table 3).

For the bacterial communities, both the abundance-based coverage estimator (ACE) and Chao1 indices decreased progressively from CK to R and RB, with significant differences among all three treatments (*p* < 0.05). The ACE index decreased by 6.14% under R and 16.31% under RB relative to CK, while the Chao1 index decreased by 6.20% under R and 16.43% under RB. The Simpson and Shannon indices were significantly reduced under RB compared with CK (*p* < 0.05). Specifically, the Shannon index decreased by 3.05% under RB, while the Simpson index declined by 0.03% under RB (*p* < 0.05, Table 3). In contrast, none of the fungal alpha diversity metrics (ACE, Chao1, Simpson, Shannon, PD_whole_tree) exhibited significant variation across the CK, R, and RB treatments (all *p* > 0.05, Table 3).

**Figure 2:**
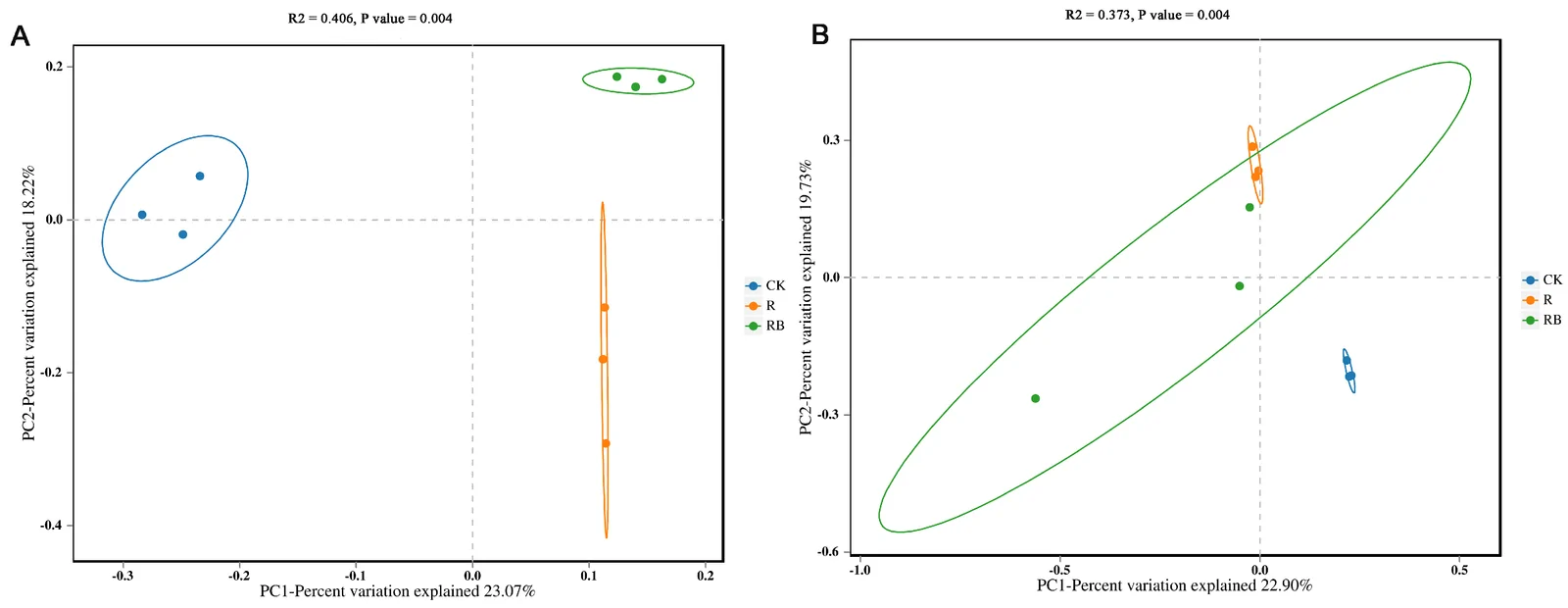
Beta diversity, assessed via principal coordinates analysis (PCoA) based on Bray–Curtis dissimilarity. (**A**) PCoA of soil bacterial communities under different treatments. (**B**) PCoA of soil fungal communities under different treatments. CK, continuous-cropping control without rapeseed biofumigation or biochar amendment; R, rapeseed biofumigation alone; RB, rapeseed biofumigation with corn-straw biochar.

Bray–Curtis-based PCoA showed treatment-associated separation in both bacterial and fungal community composition (Figure 2). For bacterial communities, PC1 and PC2 explained 23.07% and 18.22% of the variation, respectively, whereas for fungal communities, they explained 22.90% and 19.73%, respectively. PERMANOVA detected significant treatment-associated differences in both bacterial (R^2^ = 0.406, *p* = 0.004) and fungal (R^2^ = 0.373, *p* = 0.004) communities. However, PERMDISP also detected significant heterogeneity in multivariate dispersion for both bacteria (*F* = 5.962, *p* = 0.045) and fungi (*F* = 4.177, *p* = 0.039). For bacterial communities, pairwise comparisons showed a significant difference in dispersion between RB and CK (Tukey-adjusted *p* = 0.037), whereas R versus CK (*p* = 0.723) and RB versus R (*p* = 0.100) were not significant. For fungal communities, none of the pairwise dispersion comparisons were significant after Tukey adjustment (R versus CK, *p* = 0.975; RB versus CK, *p* = 0.089; RB versus R, *p* = 0.118), despite the significant overall PERMDISP result. Significant dispersion heterogeneity in both communities means that the PERMANOVA results may partly reflect differences in within-treatment dispersion and should be interpreted with caution.

### 3.5. Soil Bacterial Community Composition at Phylum and Genus Levels

**Figure 3:**
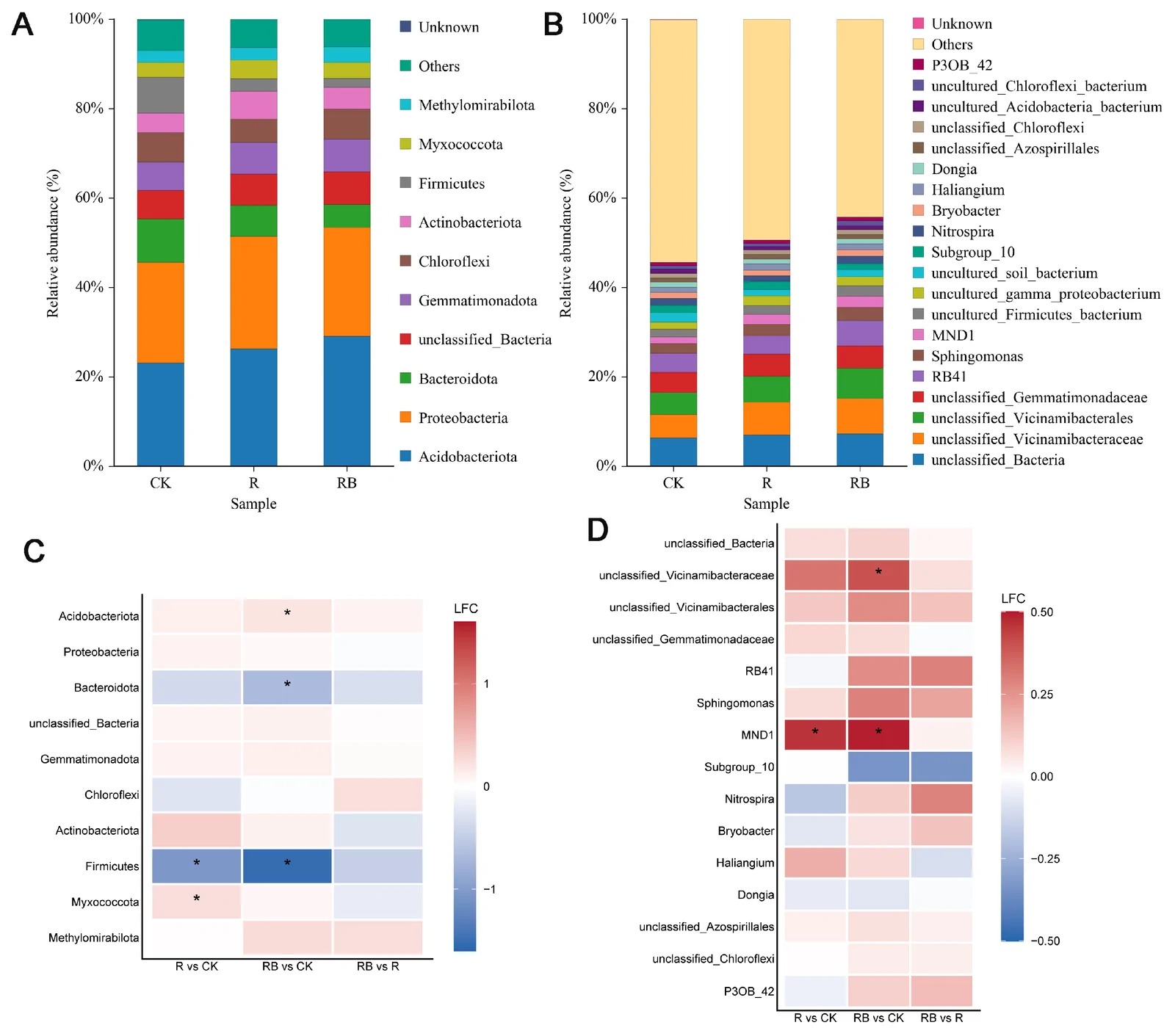
Effects of different treatments on soil bacterial community composition at the phylum and genus levels. (**A**) Relative abundance of the top 10 dominant bacterial phyla. (**B**) Relative abundance of the top 20 dominant bacterial genera. (**C**) Heatmap displaying ANCOM-BC2 log fold changes (LFCs, natural-log scale) of bacterial phyla for pairwise comparisons (R vs. CK, RB vs. CK, RB vs. R). (**D**) Corresponding heatmap of bacterial genera. Asterisks indicate significant differences (*q* < 0.05). CK, continuous-cropping control without rapeseed biofumigation or biochar amendment; R, rapeseed biofumigation alone; RB, rapeseed biofumigation with corn-straw biochar.

In total, 39 bacterial phyla, 100 classes, and 1142 genera were annotated across the rhizosphere samples. Seven dominant phyla—Acidobacteriota, Proteobacteria, Bacteroidota, Chloroflexi, Gemmatimonadota, Actinobacteriota, and Firmicutes—collectively constituted 79.5–80.7% of the total bacterial sequences (Figure 3A). At the genus level, the dominant taxa included unclassified_Bacteria, unclassified_Vicinamibacteraceae, unclassified_Vicinamibacterales, unclassified_Gemmatimonadaceae, RB41, *Sphingomonas*, MND1, uncultured_Firmicutes_bacterium, uncultured_gamma_proteobacterium, uncultured_soil_bacterium, and others (Figure 3B).

At the phylum level, RB significantly enriched Acidobacteriota (23.15% in CK vs. 29.09% in RB; LFC = +0.20, *q* = 0.029) and reduced Bacteroidota (9.72% in CK vs. 5.13% in RB; LFC = −0.67, *q* = 0.004) and Firmicutes (8.08% in CK vs. 2.03% in RB; LFC = −1.51, *q* = 0.013) relative to CK (Figure 3C). Compared with CK, the R treatment also markedly reduced Firmicutes (2.86% in R; LFC = −1.05, *q* = 3.1 × 10^−4^), while increasing Myxococcota (3.22% in CK vs. 4.18% in R; LFC = +0.25, *q* = 0.049). No phylum showed a robust significant difference between R and RB.

At the genus level, ANCOM-BC2 identified 38 and 37 genera as robustly differentially abundant in R vs. CK and RB vs. CK, respectively. Among the dominant taxa shown in the heatmap, MND1 was consistently enriched under both R and RB relative to CK (LFC = +0.46, *q* = 0.044 and LFC = +0.49, *q* = 0.032, respectively); unclassified_Vicinamibacteraceae was enriched under RB (LFC = +0.40, *q* = 0.026) (Figure 3D). No significant difference was observed in RB vs. R comparisons.

### 3.6. Soil Fungal Community Composition at Phylum and Genus Levels

**Figure 4:**
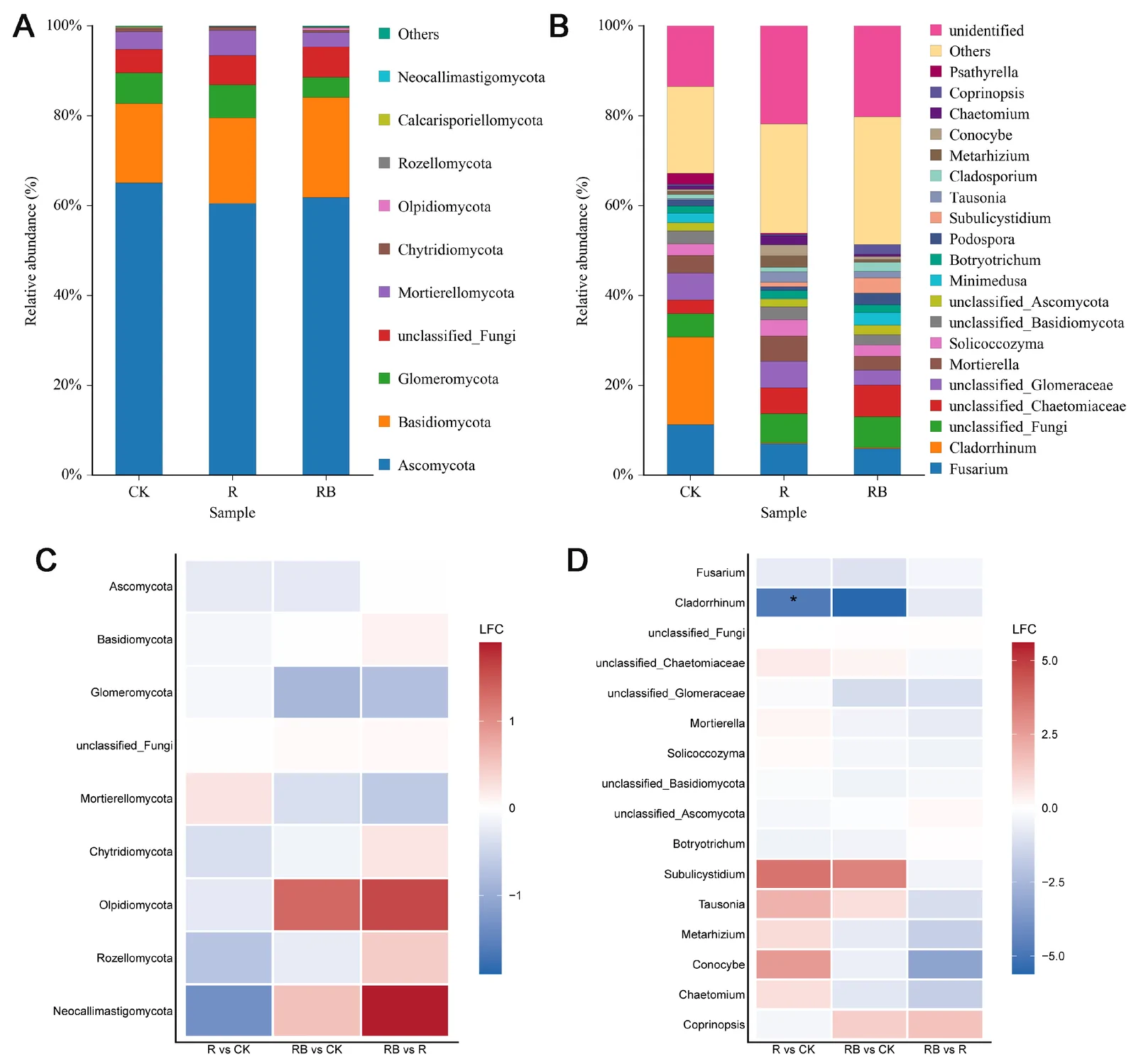
Effects of different treatments on soil fungal community composition at the phylum and genus levels. (**A**) Relative abundance of the top 10 dominant fungal phyla. (**B**) Relative abundance of the top 20 dominant fungal genera. (**C**) Heatmap displaying ANCOM-BC2 log fold changes (LFCs, natural-log scale) of fungal phyla for pairwise comparisons (R vs. CK, RB vs. CK, RB vs. R). (**D**) Corresponding heatmap of fungal genera. Asterisks indicate significant differences (*q* < 0.05). CK, continuous-cropping control without rapeseed biofumigation or biochar amendment; R, rapeseed biofumigation alone; RB, rapeseed biofumigation with corn-straw biochar.

For the fungal communities, 16 fungal phyla, 45 classes, 98 orders, 210 families, and 401 genera were identified after DADA2 denoising. The dominant fungal phyla were Ascomycota, Basidiomycota, Glomeromycota, and Mortierellomycota, accounting for 91.7–93.6% of total fungal reads (Figure 4A). The top fungal genera, displayed in Figure 4B, include *Fusarium, Cladorrhinum*, *Mortierella, Solicoccozyma, Botryotrichum, Subulicystidium*, and *Cladosporium*.

At the phylum level, no fungal phylum showed a robust significant difference among the three treatments (all *q* > 0.05; Figure 4C). Relative to CK, R was associated with a lower abundance of *Cladorrhinum* (LFC = −4.79, *q* = 0.0012; Figure 4D). The relative abundance of *Fusarium* was numerically lower under both R and RB than under CK, but neither comparison was statistically significant after ANCOM-BC2 correction (*q* ≥ 0.05). No fungal genus showed a robust significant difference between RB and CK or between RB and R.

### 3.7. Correlations of Microbial Assemblages with Soil and Plant Variables

To examine associations between rhizosphere microbial community composition and measured soil and plant variables, db-RDA, Mantel tests and Spearman’s correlation analyses were performed.

**Figure 5:**
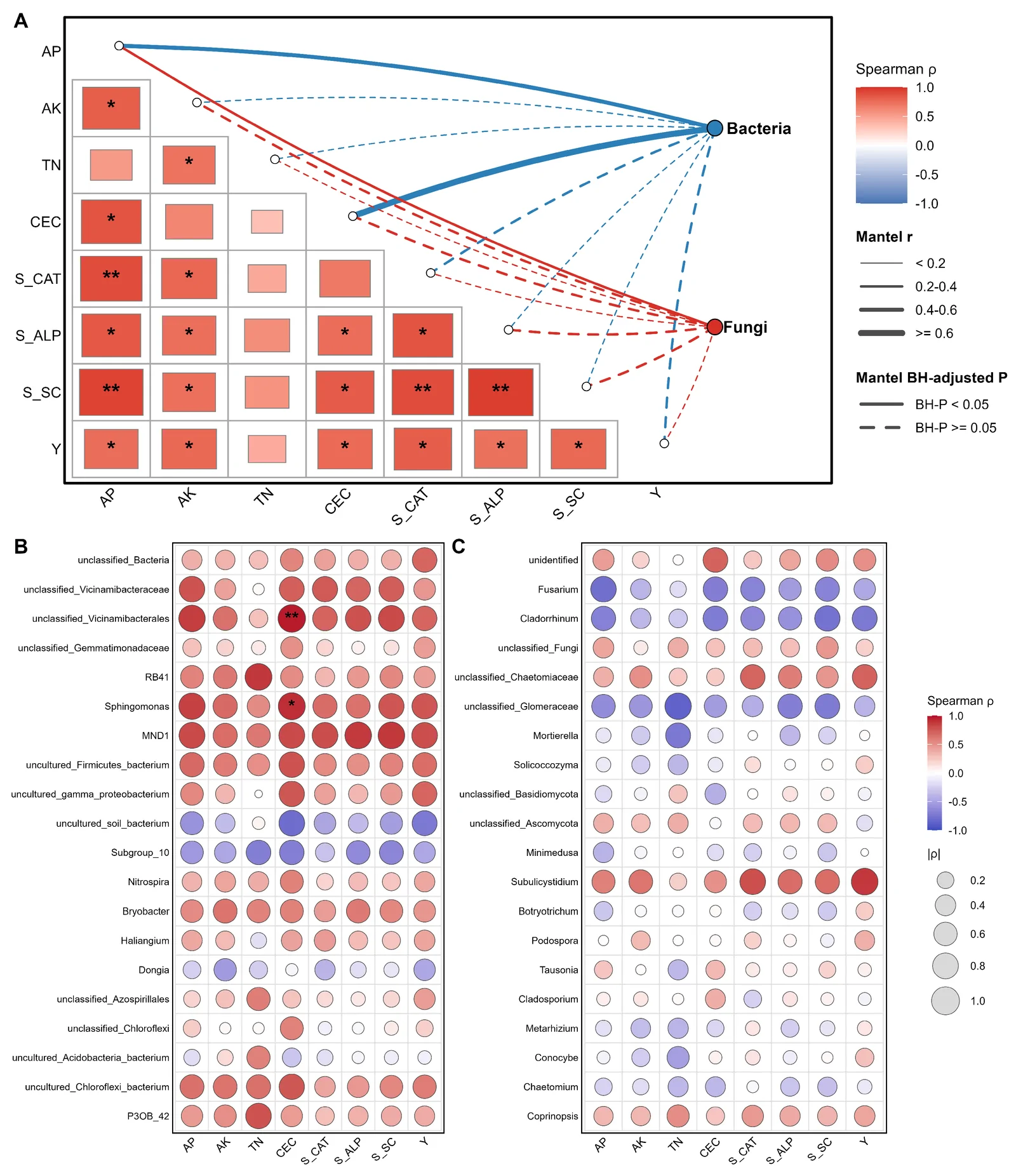
Integrative correlation analysis linking microbial communities, soil properties, enzyme activities, and flower yield. (**A**) Mantel test results quantifying correlations between community dissimilarity matrices and environmental distance matrices, combined with a pairwise Spearman correlation heatmap of the soil properties, enzyme activities, and flower yield. Spearman correlation heatmaps between the relative abundances of dominant bacterial genera and environmental variables (**B**) and between the relative abundances of dominant fungal genera and environmental variables (**C**). * FDR-*p* < 0.05, ** FDR-*p* < 0.01. AP: available phosphorus; AK: available potassium; TN: total nitrogen; CEC: cation exchange capacity; S-CAT: soil catalase; S-ALP: soil alkaline phosphatase; S-SC: soil sucrase; Y: chrysanthemum yield.

The global db-RDA model for bacterial communities was significant (*p* = 0.0286), with unadjusted R^2^ = 0.538 and a low adjusted R^2^ = 0.075. For fungal communities, the overall model was also significant (*p* = 0.0449), with unadjusted R^2^ = 0.538 and adjusted R^2^ = 0.076 (Supplementary Figure S1). Despite the significant overall models, the low adjusted R^2^ values indicate limited explanatory power of the selected variables. Mantel tests revealed that bacterial community structure was significantly correlated with soil AP and CEC, and fungal community structure was significantly correlated with AP (FDR-*p* < 0.05; Figure 5A). Spearman correlations among the soil and plant variables showed that AP, AK, CEC, S-CAT, S-ALP, S-SC, and yield formed a broadly positively correlated module, whereas TN was correlated with AK (Figure 5A).

Spearman correlation analysis was conducted on the correlated environmental factors and the relative abundances of the dominant microbial genera (Figure 5B,C). At the bacterial genus level, unclassified_Vicinamibacterales (ρ = 0.966, FDR-*p* = 0.0036) and *Sphingomonas* (ρ = 0.915, FDR-*p* = 0.0427) showed significant positive correlations with CEC. No other bacterial genera retained significant correlations after BH correction. Among the fungal genera, none of the genus-environment pairwise Spearman associations remained significant following BH correction, even though Mantel tests detected a significant association between AP and overall fungal community structure. These results indicate that the community-level association with AP was not accompanied by statistically significant associations between AP and individual fungal genera examined in this analysis.

## 4. Discussion

This field study showed that the integrated application of rapeseed biofumigation and corn-straw biochar improved selected soil fertility parameters and was associated with changes in rhizosphere microbial community structure and lower wilt disease severity in an eight-year monoculture chrysanthemum system. However, the absence of a biochar-only treatment prevents us from separating the effects of biochar itself from its interaction with biofumigation. Exploratory correlation analyses further indicated that AP and CEC were associated with variation in rhizosphere microbial community structure. Together, these observations link green soil amendments, soil biochemical status, rhizosphere microbial community modification, and chrysanthemum productivity, while the underlying causal mechanisms remain to be tested.

Our field results revealed that the R treatment significantly increased soil AP and CEC, whereas RB significantly increased soil TN, AP, AK, and CEC, as well as S-CAT, S-SC, and S-ALP activities. Previous research has shown that rapeseed green manure decomposition releases N, P, and K and increases soil nutrient content [25,26]. Biochar can also increase CEC and contribute to nutrient retention [27,28]. However, direct inputs of nutrients and organic carbon from the 30 t·ha^−1^ biochar amendment may also have contributed to the improvements observed under RB. The relative contributions of nutrient inputs and nutrient retention cannot be distinguished in the present study.

Soil enzymes serve as sensitive indicators of microbial activity and nutrient cycling processes [29]. Catalase mirrors the capacity of soil microorganisms to alleviate oxidative stress via reactive oxygen degradation; sucrase participates directly in soil carbon turnover and provides energy for microbial proliferation; and alkaline phosphatase catalyzes the hydrolysis of organic P compounds, contributing to P mineralization [30,31,32]. Here, the R treatment exerted minimal effects on soil enzyme activity, whereas the RB treatment significantly increased the activities of S-CAT, S-SC, and S-ALP. Such distinct responses may be related to biochar-induced changes in microbial microhabitats and substrate availability, which may influence soil enzyme activities and nutrient transformations [28,33,34]. The increase in S-ALP activity in the RB plots coincided with the increase in AP content, suggesting potential shifts in soil phosphorus cycling [35]. Collectively, our findings on soil nutrients and enzyme activity show that the combined application resulted in significantly higher AP and AK contents and S-SC and S-ALP activities than biofumigation alone.

The field data revealed that both R and RB had lower wilt disease indices than CK, with no significant difference between R and RB. Rapeseed tissues are rich in glucosinolates, which undergo myrosinase-mediated hydrolysis to generate volatile ITCs. This hydrolysis reaction is triggered by the mechanical maceration of plant residues and adequate soil moisture. Previous studies have reported that ITCs can disrupt fungal cell membranes, suppress spore germination, and inhibit mycelial growth, providing a possible explanation for the disease-reducing effects of biofumigation [9,36]. However, soil ITC concentrations and their effects on the causal agents of wilt disease were not measured in this experiment; therefore, this mechanism was not established here. The activity of ITCs can be constrained by their rapid volatilization and short residence time in soil [37,38]. Previous research has also shown that biochar can alter fumigant adsorption and dissipation [19,39,40], suggesting that changes in ITC retention could potentially contribute to treatment responses. Nevertheless, ITC adsorption and release were not measured in this study, and the absence of a significant difference in DI between R and RB provides no evidence that biochar enhanced disease reduction through this mechanism. Incorporation of large amounts of fresh *Brassica juncea* biomass also introduces organic matter and mineral nutrients and exerts pre-plant cover-crop effects. These inputs and effects may also have contributed to the observed treatment responses. The present design cannot distinguish their contributions from those of glucosinolate-derived ITCs.

In terms of the vegetative performance of chrysanthemum, only the RB combination significantly increased plant height and fresh flower yield. Improved nutrient availability offers one possible explanation for the observed improvement. Specifically, the RB amendment increased soil nutrient availability, particularly P and K availability, which may have supported vegetative growth and subsequent flower development in chrysanthemum [41]. RB was also accompanied by changes in rhizosphere microbial community composition and a lower wilt disease index. These changes coincided with increased plant height and fresh flower yield, but the present study did not establish causal relationships among these observations. No significant intertreatment differences were detected in stem diameter or leaf SPAD. The lack of detectable responses in these traits may partly reflect the influence of cultivar genetic background and other environmental factors [42]. These findings indicate that, under the present conditions, rapeseed biofumigation coupled with biochar improved selected soil fertility parameters and increased flower yield, while being accompanied by lower wilt disease severity, suggesting a potentially eco-friendly amelioration strategy for continuous chrysanthemum cultivation regions such as Shanxi, China, pending multi-site and multi-season validation.

The soil microbiome plays an important role in sustaining soil ecological functions and plant health. R and RB were associated with changes in microbial community composition, and RB additionally showed lower bacterial Shannon and Simpson indices than CK. For bacterial richness, bacterial ACE and Chao1 indices decreased progressively from CK to R and RB. In contrast, fungal alpha diversity did not differ significantly among treatments. PCoA combined with PERMANOVA further suggested that the treatments were associated with shifts in the bacterial and fungal community composition; however, because PERMDISP indicated significant heterogeneity of within-group dispersion, these structural differences should be interpreted with caution. These findings suggest a more evident treatment-associated response in bacterial alpha diversity than in fungal alpha diversity in this trial. The observed bacterial community changes are broadly consistent with prior research indicating that glucosinolate addition and biofumigation can alter soil bacterial communities [13,38]. Nevertheless, reduced microbial diversity does not necessarily indicate impaired ecosystem functionality. The observed community changes were accompanied by a lower wilt disease index. However, whether a functionally disease-suppressive microbiome was established requires direct experimental verification.

At the bacterial phylum level, R markedly reduced the relative abundance of Firmicutes while increasing that of Myxococcota. Firmicutes includes fast-growing copiotrophic members of genera such as *Bacillus* and *Paenibacillus* [43,44]. Suppression of susceptible members could release niches and resources for other groups. In this context, the enrichment of Myxococcota, a phylum containing predatory myxobacteria that may function as keystone bacterivores in the soil microbial food web [45], is particularly noteworthy. As biofumigation can suppress susceptible microorganisms [10], the relative enrichment of Myxococcota under R may reflect changes in competition and prey availability. Under biochar co-application, Acidobacteriota became significantly enriched, whereas Bacteroidota and Firmicutes declined relative to CK. Acidobacteriota can contribute to organic matter decomposition and nutrient cycling [46]. Previous research showed that wood sawdust biochar addition increased the relative abundance of Acidobacteriota while decreasing that of Bacteroidota [47]. The enrichment of Acidobacteriota alongside the reductions in Bacteroidota and Firmicutes under RB is broadly consistent with the oligotroph–copiotroph ecological framework [43,48]. At the bacterial genus level, unclassified_Vicinamibacteraceae was significantly enriched under the RB treatment, and MND1 was consistently enriched under both R and RB. Vicinamibacteraceae represents an oligotrophic acidobacterial lineage adapted to low-nutrient, recalcitrant-carbon conditions [49], in line with the phylum-level enrichment of Acidobacteriota. MND1 is a genus-level taxon assigned to the family Nitrosomonadaceae within Proteobacteria. Previous research reported higher MND1 relative abundance in high-yield cotton fields and positive correlations with soil total carbon, total nitrogen, and alkaline phosphatase activity [50]. MND1 was also enriched near pristine biochar under waterlogged conditions and discussed as a putative ammonia-oxidation-associated taxon [51]. Its enrichment under both R and RB may therefore reflect responses to altered rhizosphere conditions, with potential relevance to nitrogen transformations. Taken together, these community changes suggest potential shifts in bacterial resource utilization, microbial trophic interactions, and nitrogen transformations. Their consequences for soil fertility and pathogen suppression warrant verification in future studies.

The fungal community showed fewer robust taxon-level responses to the treatments than the bacterial community. At the genus level, *Cladorrhinum* showed a significantly lower abundance under R than under CK, whereas no robust difference was detected between RB and CK or between RB and R. Members of *Cladorrhinum* are commonly found in soil and plant debris, and some strains have been reported to colonize plant roots and exhibit plant-beneficial traits [52,53]. The relative abundance of *Fusarium* was numerically lower under both R and RB than under CK, although neither difference was statistically significant. This trend coincided with the lower disease indices under R and RB. However, genus-level ITS profiles cannot distinguish pathogenic from non-pathogenic *Fusarium* lineages, and changes in relative abundance do not establish changes in absolute population size. Targeted detection of pathogenic *Fusarium* lineages and absolute quantification would be needed to assess changes in pathogen populations.

Furthermore, bacterial community structure was significantly associated with soil AP and CEC, whereas fungal community structure was significantly associated with AP. Among dominant bacterial genera, only unclassified_Vicinamibacterales and *Sphingomonas* exhibited significant positive correlations with CEC, and no dominant fungal genus retained significant correlations with any measured environmental variable. This pattern suggests that the observed community-level association with AP may reflect collective changes across multiple microbial taxa, rather than strong AP associations detectable at the level of individual dominant genera. Given synchronous treatment-driven changes in AP, AK, CEC, enzyme activities, and yield, these variables are heavily confounded, and our correlational dataset cannot resolve causal relationships. Collectively, the treatments were accompanied by changes in rhizosphere microbial community composition, selected soil properties and enzyme activities, together with lower wilt disease severity and higher chrysanthemum yield under the conditions of this field trial.

However, this study had several limitations. First, the experimental design did not include a biochar-only treatment; therefore, the interaction between biofumigation and biochar could not be formally tested, and the observed effects of the combined treatment cannot be attributed to synergy, additivity, or biochar alone. Second, our current design lacks a biomass-matched non-biofumigant green-manure control. The rapeseed biofumigation treatments received large quantities of incorporated fresh brassica biomass, bringing confounding green-manure organic-matter inputs, nutrient mineralization, and pre-plant cover-crop effects that are absent in the CK group. Consequently, we cannot exclusively attribute treatment differences to glucosinolate-derived isothiocyanates. Third, the field trial covered only a single chrysanthemum growing season; long-term monitoring is required to clarify the persistent effects and cumulative impacts of the combined amendment on soil microbiomes. Fourth, the adsorption and slow-release mechanisms of ITCs on biochar were deduced indirectly, without direct experimental evidence of adsorption kinetics. Additionally, only amplicon sequencing was used to profile community shifts, whereas metagenomic and metabolomic analyses were not used to decipher microbial metabolic reprogramming. Our future work will integrate multiomics technologies to dissect the metabolic pathways of disease-suppressive microbial taxa, and we will conduct gradient field trials covering diverse biochar feedstocks and application dosages to formulate standardized green cultivation regimes for tea chrysanthemum.

In conclusion, this single-season field trial on eight-year continuously monocropped brown soil in Wenxi County, Shanxi Province, showed that rapeseed biofumigation combined with corn-straw biochar (RB) improved selected soil fertility parameters and increased chrysanthemum flower yield relative to CK. Both R and RB were accompanied by changes in rhizosphere microbial community composition and significantly lower wilt disease indices than CK, with no significant difference in disease index between R and RB. These findings do not establish a causal link between microbial community changes and disease reduction or yield improvement. Multi-site, multi-season field trials incorporating a biochar-only treatment within a factorial design, together with mechanistic investigations, are needed to assess the broader applicability of these findings.

## Figures and Tables

**Table 1:** Effects of biofumigation combined with biochar on soil physicochemical properties.

Group	pH	Available Phosphorus (mg·kg^−1^)	Available Potassium (mg·kg^−1^)	Organic Matter (g·kg^−1^)	Total Nitrogen (g·kg^−1^)	Alkali-Hydrolyzable Nitrogen (mg·kg^−1^)	Cation Exchange Capacity (cmol_c·kg^−1^)
CK	8.20 ± 0.04 ^a^	21.20 ± 0.50 ^c^	212.00 ± 6.08 ^b^	14.50 ± 0.36 ^a^	1.02 ± 0.02 ^b^	67.90 ± 1.04 ^a^	18.50 ± 0.26 ^b^
R	8.13 ± 0.03 ^a^	25.67 ± 1.04 ^b^	216.33 ± 1.53 ^b^	14.87 ± 0.31 ^a^	1.00 ± 0.02 ^b^	68.53 ± 1.85 ^a^	19.77 ± 0.15 ^a^
RB	8.14 ± 0.04 ^a^	29.83 ± 0.25 ^a^	246.67 ± 5.86 ^a^	15.00 ± 0.20 ^a^	1.07 ± 0.01 ^a^	69.77 ± 1.10 ^a^	20.10 ± 0.35 ^a^

Note: CK = continuous-cropping control without rapeseed biofumigation or biochar amendment; R = rapeseed biofumigation alone; RB = the combined application of rapeseed biofumigation and corn-straw biochar. Data are presented as means ± standard deviation (n = 3). Different lowercase letters within each column denote significant differences at *p* < 0.05 based on Duncan’s multiple range test.

**Table 2:** Wilt disease index (DI) and control efficacy (CE) under different soil amendments.

Group	DI (%)	CE (%)
CK	8.67 ± 1.15 ^a^	-
R	2.67 ± 0.58 ^b^	69.23 ± 6.66
RB	2.33 ± 0.58 ^b^	73.08 ± 6.66

Note: CK = continuous-cropping control without rapeseed biofumigation or biochar amendment; R = rapeseed biofumigation alone; RB = the combined application of rapeseed biofumigation and corn-straw biochar. Data are presented as means ± standard deviation (n = 3). Different lowercase letters in the DI column indicate significant differences among treatments based on an analysis of variance including treatment and block, followed by Tukey’s HSD test (*p* < 0.05). CE is a descriptive indicator derived from DI and was not subjected to a separate significance test.

**Table 3:** Alpha-diversity indices of rhizosphere bacterial and fungal communities.

Community	Group	ACE	Chao1	Simpson	Shannon	PD_Whole_Tree	Coverage (%)
Bacteria	CK	2989.70 ± 40.04 ^a^	2981.77 ± 39.96 ^a^	0.9987 ± 0.0001 ^a^	10.50 ± 0.05 ^a^	37.24 ± 0.34 ^a^	99.95 ± 0.00 ^a^
	R	2806.02 ± 12.51 ^b^	2796.97 ± 10.36 ^b^	0.9987 ± 0.0002 ^a^	10.39 ± 0.05 ^a^	37.03 ± 0.48 ^a^	99.95 ± 0.02 ^a^
	RB	2501.94 ± 130.72 ^c^	2491.74 ± 130.82 ^c^	0.9984 ± 0.0000 ^b^	10.18 ± 0.05 ^b^	36.09 ± 0.93 ^a^	99.94 ± 0.01 ^a^
Fungi	CK	549.13 ± 43.44 ^a^	547.30 ± 46.87 ^a^	0.9476 ± 0.0208 ^a^	6.30 ± 0.30 ^a^	74.90 ± 5.69 ^a^	99.95 ± 0.03 ^a^
	R	521.29 ± 22.17 ^a^	523.34 ± 20.39 ^a^	0.9743 ± 0.0135 ^a^	6.92 ± 0.41 ^a^	62.05 ± 6.71 ^a^	99.95 ± 0.02 ^a^
	RB	536.17 ± 139.56 ^a^	528.40 ± 150.87 ^a^	0.9744 ± 0.0024 ^a^	6.87 ± 0.36 ^a^	75.11 ± 12.55 ^a^	99.94 ± 0.01 ^a^

Note: CK = continuous-cropping control without rapeseed biofumigation or biochar amendment; R = rapeseed biofumigation alone; RB = the combined application of rapeseed biofumigation and corn-straw biochar; ACE = abundance-based coverage estimator. Data are presented as means ± standard deviation (n = 3). Distinct lowercase letters among treatments within each column for the same community indicate significant differences (*p* < 0.05).

## Data Availability

Sequence data that support the findings of this study have been deposited in NCBI Sequence Read Archive (SRA) with the primary accession code PRJNA1521150.

## Supplementary Materials

The following supporting information can be downloaded at: https://www.mdpi.com/article/10.3390/microorganisms14102383/s1, Figure S1: Distance-based redundancy analysis (db-RDA) showing the relationships between rhizosphere microbial community composition and selected soil/plant variables. (A) Bacterial community; (B) Fungal community.
