# Supplementary material for: Rapeseed Biofumigation Combined with Biochar Mitigates Continuous-Cropping Obstacles in Chrysanthemum via Soil Improvement, Rhizosphere Microbial-Community Modification, and Wilt Disease Reduction
Source: Microorganisms. 2026 Oct 18;14(10):2383. doi: 10.3390/microorganisms14102383 (PMC13707554; doi:10.3390/microorganisms14102383)
Supplement: Supplementary file 1 [file microorganisms-14-02383-s001.zip › microorganisms-4531535-supplementary.pdf]

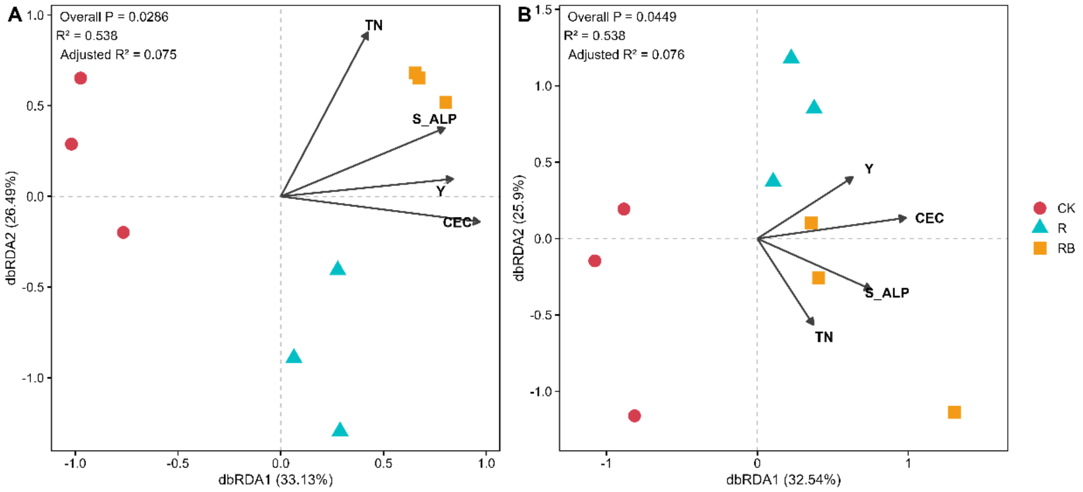

Figure S1 Distance-based redundancy analysis (db-RDA) showing the relationships between rhizosphere microbial community composition and selected soil/plant variables. (A) Bacterial community; (B) Fungal community.
